# Advancement in Functionalized Electrospun Nanofiber-Based Gas Sensors: A Review

**DOI:** 10.3390/s25164896

**Published:** 2025-08-08

**Authors:** Yanjie Wang, Zhiqiang Lan, Jie Wang, Kun Zhu, Jian He, Xiujian Chou, Yong Zhou

**Affiliations:** 1 School of Future Science and Engineering, Soochow University, Suzhou 215222, China; yjwang0220@suda.edu.cn (Y.W.);; 2 Science and Technology on Electronic Test and Measurement Laboratory, School of Instrument and Electronics, North University of China, Taiyuan 030051, China; 3 Key Laboratory of Optoelectronic Technology and Systems of Ministry of Education of China, Chongqing University, Chongqing 400044, China

**Keywords:** electrospinning nanofibers, gas sensors, functional strategy, metal oxides, carbon materials, conductive polymers

## Abstract

In recent years, electrospinning technology has sparked a revolution in the nanoengineering of gas-sensing materials. Nanofibers based on metal oxide semiconductors, carbon materials, or conductive polymers prepared by the electrospinning process have exhibited inspiring properties, including a large specific surface area, porous structure, and nice stability, with bright application prospects in advanced gas sensors. Meanwhile, the increasingly expanding applications of gas sensors, such as the Internet of Things (IoT), the food industry, disease diagnosis, etc., have raised higher sensor performance requirements. To further enhance the gas-sensing performance of nanofibers, the scheme of functionalized nanofiber strategies, either in electrospinning or post-treatment, has been proposed and verified. This review systematically summarized the nanostructures, gas-sensing properties, and functional mechanisms of modified nanofibers. Additionally, the perspectives and challenges regarding electrospun nanofibers for gas sensing were discussed.

## 1. Introduction

Gas sensors play a critical role in fields such as the Internet of Things (IoT), environmental monitoring, healthcare diagnostics, and food safety [1,2,3,4,5]. Among various types of gas sensors, chemiresistive gas sensors have attracted considerable attention due to their remarkable advantages, including high sensitivity, sub-ppm level detection limits, cost-effective fabrication processes, and easy integration [6,7,8,9,10]. With the widespread application of chemiresistive gas sensors, emerging application scenarios are imposing more demands on their performance, such as ultrahigh sensitivity, rapid response/recovery, excellent selectivity, and long-term stability [11,12]. Flexible gas sensors can be utilized in wearable human devices for sustained physiological monitoring, where the mechanical properties of the flexible sensors critically determine the reliability [13]. Gas-sensing materials serve as the fundamental determinant of sensor performance, where their strategic design and innovation constitute the cornerstone for developing next-generation high-performance gas sensors. Thereinto, metal oxides (SnO_2_, ZnO), carbon nanostructures (graphene, carbon nanotubes), and conductive polymers (polyaniline, polypyrrole) were widely applied [14,15]. The sensing properties of these materials rely on three critical factors: (1) receptor function, (2) transducer function, and (3) utility factor, which regulate the gas molecule adsorption, signal conversion, and gas diffusion, respectively [16].

Traditional chemiresistive gas sensors based on bulk materials widely suffer from limitations such as low specific surface area, limited sensitivity, poor reversibility, and sluggish adsorption/desorption kinetics [17]. In contrast, 1D nanofiber materials possess superior sensing performance due to unique advantages, including high porosity, restricted electronic transmission dimensions, tunable morphology, and scale fabrication [18]. Cho et al. investigated the NO_2_ sensing performance of SnO_2_ hollow nanofibers with diameters of 300–500 nm by electrospinning and SnO_2_ thin film with a thickness of 15–20 nm by RF-sputtering, respectively [19]. The SnO_2_ hollow nanofibers exhibited a promoted response of 81.4 to 2 ppm NO_2_ compared to that (19.9) of the SnO_2_ thin film. The enhancement resulted from the greater specific surface area and greater space charge modulation depth of the 1D hollow nanofibers. Conventional nanofiber fabrication techniques, such as chemical vapor deposition, sol–gel methods, and template-assisted synthesis, enable precise control over material composition and morphology, but suffer from limitations including high energy consumption and reliance on sophisticated equipment [20]. In contrast, electrospinning technology stands out due to its operational simplicity and low cost, allowing facile regulation of fiber morphology and composition [21]. Specifically, electrospun nanofibers possess a uniform 1D structure with a high surface area, which significantly enhances gas adsorption capacity and accelerates adsorption/desorption kinetics of the sensing layer. Furthermore, the physicochemical properties of nanofibers can be precisely tailored by optimizing electrospinning and post-treatment parameters (solution concentration, applied voltage, collector distance, calcination temperature), enabling highly sensitive and selective detection toward target gases. Morais et al. prepared a sensitive NO_2_ sensor based on WO_3_ nanofibers, which were synthesized by electrospinning and following calcination [22]. The thickness and grain size of WO_3_ nanofibers were dependent on the calcination heating rate and heating temperatures, and thus regulating the gas-sensing performance. The optimized WO_3_ nanofibers, calcined at 500 °C with a heating rate of 10 °C/min, presented a high response of 15,000 toward 25 ppm NO_2_.

Although the technology of electrospun nanofibers has significantly promoted the gas-sensing performance of sensitive materials, the intrinsic limitations of these materials themselves still frustrate their further application. For instance, metal oxides suffered from high operating temperatures and poor selectivity [23]; conductive polymers were limited by weak stability and poor reversibility [24]; carbon nanofibers lacked specific adsorption behavior to various gases and had insufficient sensitivity to target gas [25,26]. Despite photoirradiation [27] and plasma treatment [28] being effective strategies to overcome these obstacles, they still faced critical limitations such as material degradation under prolonged exposure, high operational costs, and insufficient selectivity due to non-specific surface activation, ultimately restricting their scalability and long-term stability in practical applications [29].

Utilizing surface and interface engineering strategies to functionalize the gas-sensitive materials is one facile method to promote the sensing performance of sensors. The functionalized gas-sensitive materials could overcome the limitations inherent in single-component materials, where the synergistic interaction between constituents significantly enhances gas-sensing performance [30,31]. Recently, composite nanofibers fabricated via co-electrospinning or post-treatment strategies have garnered significant attention [32,33]. For instance, doping with noble metal could improve the sensitivity and accelerate response speed by chemical sensitization and electronic sensitization [34]. Metal oxides could be constructed as heterojunctions with host nanofiber materials, thus regulating the interface potential energy barriers [35]. These composites synergistically integrate functional components into electrospun nanofibers, offering enhanced gas adsorption capacities, catalytic activity, and electrical properties compared to pristine nanofibers.

This review comprehensively summarized recent advancements of functionalized electrospun nanofibers in gas sensors. Initially, the gas-sensing materials derived by electrospinning technology were introduced, including the synthesis process and gas-sensing mechanism of metal oxides, carbon materials, and polymers. Then, the gas sensors based on functionalized nanofibers were discussed in detail, including the construction method, enhancement mechanism, and sensing performance. Furthermore, the emerging challenges and outlined future directions of composite nanofibers for gas sensing were presented to accelerate the practical application.

## 2. Overview of the Technology of Electrospinning Nanofibers

**Figure 1:**
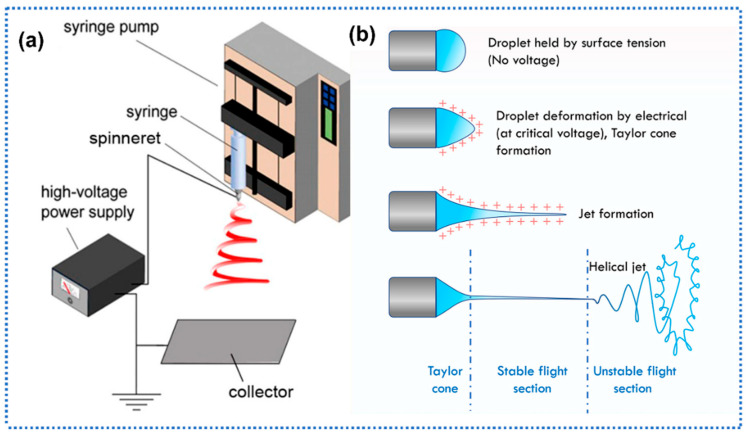
(**a**) Schematic illustration of the setup for electrospinning, reprinted with permission from Ref. [37]. Copyright 2017, The American Chemical Society. (**b**) Mechanism of electrospinning, reprinted with permission from Ref. [38]. Copyright 2024, Cell Press.

Electrospinning technology is a versatile and efficient technique for fabricating nanofibers characterized by remarkably high specific surface areas and precisely tunable morphologies. A standardized electrospinning apparatus consists of four functional modules [36]: (1) a high-voltage power supply, (2) a grounded collector designed for nascent fiber acquisition, (3) a precision syringe pump system with a metallic needle, and (4) a flow-control mechanism regulating polymer solution delivery, as shown in Figure 1a.

The operation of electrospinning starts with the establishment of an intense electrostatic field between the needle tip and the collector. The syringe pump delivers polymer solution at controlled flow rates, generating charged droplets that remain suspended at the needle orifice. At critical voltage thresholds, the balance between electrostatic forces, surface tension, and gravitational pull induces cone-jetting phenomena, forming characteristic “Taylor cones” (Figure 1b).

Subsequent ejection of charged fluid filaments occurs as micro-jets directed toward the collector. During mid-air transport, non-uniform charge distribution within the jets induces dynamic instability under sustained electric fields, manifesting as whipping, bending, and progressive elongation. This morphological evolution is synergistically facilitated by concurrent solvent evaporation, leading to continuous jet thinning through successive bifurcations. The process culminates in electrostatic stretching and solidification of polymer matrices, ultimately depositing hierarchically structured micro/nanofibers with submicron-scale diameters onto the collector substrate.

## 3. Preparation and Sensing Mechanism of Electrospun Nanofiber Gas Sensors

### 3.1. Synthesis of Gas-Sensing Materials Using Electrospinning

**Figure 2:**
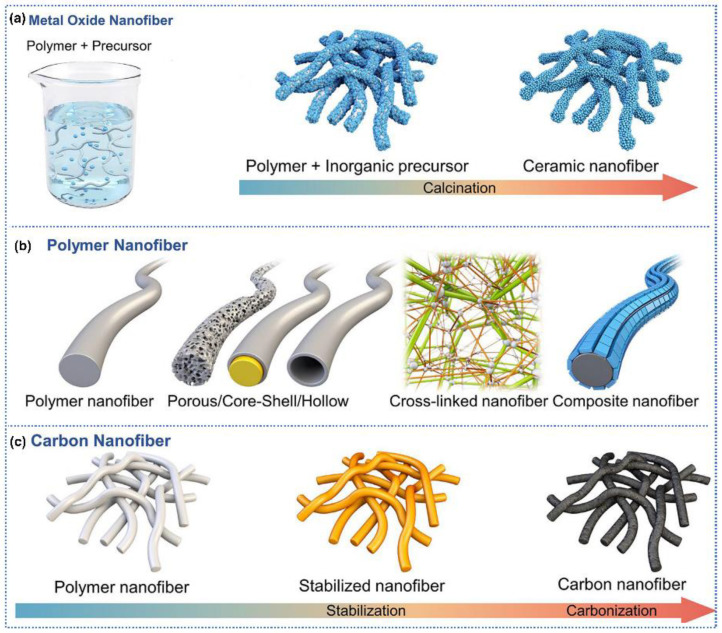
Schematic illustration of gas-sensitive materials synthesized by electrospinning: (**a**) metal oxide nanofibers, (**b**) polymer nanofibers, (**c**) carbon nanofibers. Reprinted with permission from Ref. [49]. Copyright 2025, Wiely.

Regarding the gas-sensitive materials synthesized by electrospinning, metal oxides, polymers, and carbon nanofibers were widely investigated [39,40]. As for the metal oxide nanofibers. Electrospinning technology, through optimizing the type of precursors and precise adjustment of process parameters supplemented by heat treatment, provides a versatile and scalable platform for producing metal oxide nanofibers (Figure 2a) [41]. Specifically, a polymer solution containing metal ion precursors (metal alkoxides or metal salts) was electrospun into nanofibers. Subsequently, these fibers undergo calcination at elevated temperatures, during which the polymer oxidatively decomposes and metal oxide crystals grows, ultimately forming metal oxide nanofibers with well-defined morphology, uniform diameter, and tunable porosity. By regulating precursor composition, solution properties, and calcination parameters, precise modulation of the crystallinity, specific surface area, and size of the metal oxide nanofibers can be achieved [42].

Electrospinning fabricates polymer nanofibers with uniform diameters and high aspect ratios, exhibiting exceptional structural consistency alongside scalable processability (Figure 2b). The critical advantages reside in facile tunability, where precise adjustments to key process parameters (polymer concentration, solution viscosity, applied voltage, and needle-collector distance) enable accurate control of mechanical characteristics. In addition, hollow or porous and cross-linked polymer nanofibers could be achieved by post-treatment [43,44]; core–shell nanofibers could be obtained via coaxial spinning [45]; composite nanofibers could be synthesized by incorporating multiple polymers or nanoparticles [46].

The carbon nanofiber electrospinning process begins with the polymer precursors, followed by two thermal treatment stages, involving stabilization and carbonization (Figure 2c). Stabilization is performed at 200–500 °C under an oxidative environment, where the polymer chains are chemically modified and strengthened [47]. The subsequent carbonization process occurs under an inert atmosphere at elevated temperatures (600–3000 °C), where the polymeric matrix transforms into nanofibers with either graphitic or amorphous microstructures, which is related to the thermal treatment temperature and annealing duration [48].

### 3.2. Electrospinning Parameters

The electrospinning process is critically regulated by a set of key operational parameters, which collectively dictate fiber morphology and resultant properties by modulating electrostatic interactions, solution rheology, and jet dynamics [50]. This section systematically elucidates the impact mechanisms of these principal variables:

(1) Applied voltage

The magnitude of the applied voltage directly governs the electric field gradient and charge density distribution. Within the critical voltage window (typically 10–30 kV), elevated voltage enhances electrostatic repulsion forces, thereby overcoming solution surface tension to induce significant jet stretching [51]. This process results in an exponential decrease in fiber diameter. However, exceeding critical thresholds induces corona discharge due to charge accumulation, disrupting jet stability and promoting bead formation through fragmentation. This transition is mechanistically attributed to an excessive charge density disrupting the dynamic charge migration equilibrium necessary for continuous jetting.

(2) Solution concentration and viscosity

Polymer concentration fundamentally dictates solution viscosity and rheological behavior, critically influencing fiber continuity [52]. Suboptimal concentrations yield insufficient entanglement, allowing surface tension to dominate and form discontinuous droplets or beaded fibers. Optimal concentrations establish a balance between viscosity and conductivity, facilitating the stable formation of a Taylor cone and enabling uniform solvent evaporation for the production of smooth, continuous fibers. Conversely, excessive concentrations engender high viscoelasticity within the solution, impeding jet elongation during flight and leading to the formation of fibers with irregular, rough surfaces. These concentration-dependent effects stem fundamentally from variations in polymer chain entanglement governed by the Huggins coefficient, coupled with interfacial Marangoni effects during phase separation. Zhang et al. prepared an ethanol sensor based on electrospun SnO_2_ nanofibers and investigated the effect of PVA concentration on the morphology of SnO_2_ fibers [53]. With the increase of the concentration of PVA from 6% to 8%, the average diameter of the SnO_2_ nanofibers increased. Moreover, the nanofibers were not continuous with the PVA concentration of 8% due to the large viscoelastic force. In addition, the fiber intensity decreased with the increase in PVA concentration. Hence, the solution with 6% PVA was utilized to prepare thin and uniform fibers.

(3) Solution flow rate

The flow rate, precisely controlled by a syringe pump, determines the mass flux of polymer solution to the Taylor cone and consequently influences jet residence time under the electric field [54]. Insufficient flow rates, while potentially promoting solvent evaporation, carry the risk of needle tip clogging. Conversely, excessively high flow rates perturb the electrostatic charge balance at the jet apex, inducing jet splaying or splitting. Moreover, empirical observations indicate a near-linear increase in average fiber diameter with increasing flow rate, which is attributed to the direct scaling of polymer mass deposition per unit time with the jet’s volumetric flow rate and consequently its cross-sectional area during solidification.

(4) Tip-to-collector distance

The distance separating the spinneret tip and the collector (typically 10–30 cm) is a key determinant of the jet flight time and solvent evaporation kinetics. Insufficient distances curtail flight time, leading to incomplete solvent evaporation and resulting in the deposition of wet fibers that fuse upon contact [51]. Increasing the distance prolongs jet whipping and extensional deformation, potentially generating finer fibers; however, it simultaneously enhances the likelihood of secondary jet instabilities and breakup. An overly extended distance also compromises fiber collection efficiency due to the wider spatial dispersion of the jet.

The above electrospinning parameters were critical for the morphology and structures of nanofibers, and further affected the gas-sensing property. Khomarloo et al. optimized the structural dimension of electrospinning ZnO nanofibers to enhance the NO sensing performance [54]. The effect of voltage, feed rate, and tip-to-collector distance on nanofibers was investigated initially. The nanofibers with optimized parameters (voltage: 18 kV, feed rate: 200 μL/h, distance: 20 cm) exhibited a more porous structure with a high specific surface area of 76.76 m^2^/g and a diameter of 0.16 μm. And the sensors based on optimized nanofibers showed the best response to NO of 1 ppm and 500 ppb. Moreover, the increase in ZnO nanofibers diameter resulted in response decrease toward NO, owing to the smaller diameter promoting the surface area and active adsorption sites.

### 3.3. Gas-Sensing Mechanism of Pristine Electrospinning Nanofibers

**Figure 3:**
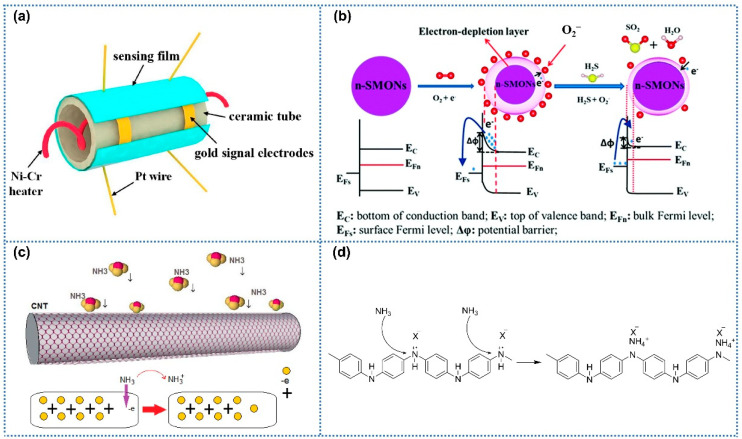
(**a**) Schematic illustration of chemiresistive gas sensor, Reprinted with permission from Ref. [59] Copyright 2018, Elsevier. (**b**) Gas-sensing mechanism of n-type metal oxides semiconductors, Reprinted with permission from Ref. [60]. Copyright 2018, The Royal Society of Chemistry. (**c**) Gas-sensing mechanism of carbon nanotubes, Reprinted with permission from Ref. [41] Copyright 2014, MDPI. (**d**) Gas-sensing mechanism of PANI, Reprinted with permission from Ref. [61] Copyright 2007, MDPI.

Figure 3a exhibited the typical structure of chemiresistive gas sensors, including the sensing layer, ceramic tubes, heating electrodes, and signal electrodes. As the critical part of gas sensors, currently, electrospinning gas-sensitive materials mainly include metal oxides, conductive oxides, and carbon materials [19,55,56]. For metal oxides, the widely accepted gas sensor mechanism involves surface redox reactions (Figure 3b) [57]. When exposed to air, oxygen molecules adsorb onto the metal oxide surface and extract electrons from its conduction band, forming oxygen species (O_2_^−^/O^−^), with the oxygen species types dependent on the operating temperature [58]. Upon exposure to reducing gases, n-type metal oxide semiconductors react with pre-adsorbed oxygen species, releasing electrons into the conduction band and increasing the electrical conductivity of the sensing layer. In contrast, p-type semiconductors exhibit opposite conductivity trends upon interacting with reducing gases. Unlike metal oxides requiring oxygen participation in gas–solid reactions, carbon-based materials and conductive polymers directly react with target gases through charge transfer mechanisms (Figure 3c,d) [40,41], thus altering the electrical conductivity of the sensitive layer without relying on atmospheric oxygen.

To assess the performance of gas sensors, the essential indicators could be summarized as: response, response/recovery times, operation temperature, and limit of detection (LoD). The response (S) is defined as S = R_g_/R_a_ or S = (R_a_ − R_g_)/R_a_ for reducing gas and S = R_a_/R_g_ or S = (R_a_ − R_g_)/R_a_ for oxidizing gas, where R_a_ and R_g_ separately represent the stable resistance of the as-prepared sensor in air and target gas. The response time (t_res_) is defined as the duration required for the resistance of sensors to reach 90% of the total resistance change when exposed to the target gas. The recovery time (t_rec_) refers to the time needed for the resistance to return to 90% of its baseline value after the target gas is removed. To assess the enhancement effects of the functionalization strategy on the gas-sensing performance of electrospun nanofibers, the ratio of the response (S_F_) of functionalized sensors to that (S_N_) of non-functionalized sensors (S_F_/S_N_) is employed as a performance enhancement metric in this study.

## 4. Gas Sensors Based on Metal Oxide (MO) Nanofibers

Metal oxides are currently the most widely used gas-sensitive materials due to their high sensitivity, fast response/recovery speed, and good stability [62,63]. Therefore, the preparation of metal oxide nanofibers for gas-sensitive materials via the electrospinning technique has attracted significant attention [64]. As for the synthesis of metal oxide nanofibers, a homogeneous precursor solution containing metal salts and polymers was prepared initially. Under a high-voltage electric field (10–30 kV), the solution is stretched into jets and collected as a fibrous membrane. Subsequently, high-temperature calcination (400–800 °C) was performed to remove organic components and convert metal salts into oxides, ultimately yielding uniform metal oxides nanofibers [65].

According to the difference in major carriers, the metal oxide semiconductors could be divided into n-type (ZnO, SnO_2_, TiO_2_, WO_3_, In_2_O_3_) and p-type (CuO, NiO, Co_3_O_4_, and Nb_2_O_3_) [66]. Kim et al. reported a highly sensitive NO_2_ sensor based on n-type SnO_2_ hollow nanofibers by electrospinning [19]. The SnO_2_ nanofibers exhibited a high response of 81.4 (R_g_/R_a_) toward 2 ppm NO_2_, surpassing that of planar SnO_2_ thin films (R_g_/R_a_ = 19.9) at 300 °C. The enhanced NO_2_-performance could be attributed to the more accessible active sites and the larger space charge modulation depth with the 1D hollow nanofibers. Lee et al. prepared a highly sensitive and selective ethanol sensor based on p-type Co_3_O_4_ nanofibers [67]. The Co_3_O_4_ nanofibers prepared by heat treatment of as-spun precursor fibers at 500 °C showed a high response of 51.2 to 100 ppm ethanol and short response/recovery times of 7.9/22.7 s at 301 °C. It could be found that metal oxides always require operation at elevated temperatures to supply sufficient activation energy for surface redox reactions and overcome carrier transport barriers [68]. However, high operation temperatures lead to significant bottlenecks: (1) accelerating grain growth that severely degrades long-term stability of sensors, and (2) increasing energy consumption for sustained operation [69]. Furthermore, single-metal-oxide-based gas sensors demonstrated detection limits at ppm or sub-ppm levels, thereby failing to achieve highly sensitive detection for trace gases at ppb levels [70]. To overcome these barriers that metal oxide nanofibers faced, functionalization method on the metal oxide nanofibers has been verified as one facile strategy [29].

### 4.1. Gas Sensors Based on Noble Metal-Doped Metal Oxides

Noble metals (Pd, Pt, Au, etc.) are widely applied in surface modifications of gas-sensing materials to enhance sensor sensitivity, due to their chemical sensitization and electronic sensitization effects [71]. With the modification of noble metals, the adsorption energy of metal oxides would be reduced, and more target gas molecules would be adsorbed on the sensing layer [72]. In addition, electronic sensitization would result in the redistribution of charge carriers, accelerating the exchange of charge between the sensing layer and the target gas [73]. Moreover, chemical sensitization would reduce the activation energy of the redox reaction and thus decrease the operation temperature [73].

**Figure 4:**
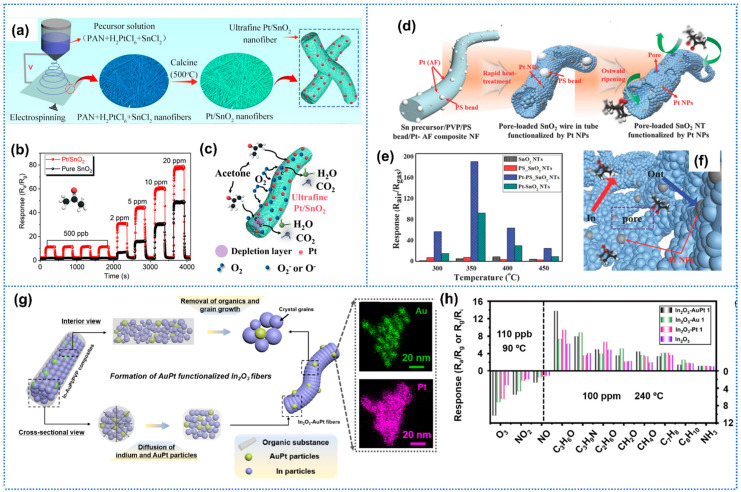
(**a**) Illustration of the synthesis process of Pt-SnO_2_ nanofibers; (**b**) The dynamic response curves of Pt-SnO_2_ and SnO_2_ toward acetone; (**c**) Schematic illustration of the acetone sensing mechanism, Reprinted with permission from Ref. [74] Copyright 2023, Elsevier. (**d**) Schematic illustration of Pt load porous SnO_2_ nanofibers; (**e**) Temperature-dependent acetone response of different sensors; (**f**) schematic illustration of the sensing mechanism for Pt-PS-SnO_2_, Reprinted with permission from Ref. [76] Copyright 2016, Wiley. (**g**) Schematic diagram of In_2_O_3_ nanofibers functionalized with AtPt particles; (**h**) Response of sensors based on pristine In_2_O_3_, In_2_O_3_-AuPt, In_2_O_3_-Au, and In_2_O_3_-Pt toward different gases, Reprinted with permission from Ref. [81] Copyright 2022, The American Chemical Society.

Cui et al. prepared Pt nanoparticles doped with ultrafine SnO_2_ mesopore nanofiber for acetone detection by an electrospinning technique and annealing process, as shown in Figure 4a [74]. The Pt-SnO_2_ sensors exhibited superior acetone-sensing performance than pure SnO_2_ sensors, including a high response of 31.2 to acetone of 2 ppm, rapid response/recovery times of 13/23 s, and a low detection limit of 100 ppb (Figure 4b). The enhanced performance could be attributed to the catalytic activity of Pt, which facilitated the conversion rate of adsorbed oxygen molecules to ions, speeding up the reaction between acetone and the sensitive layer (Figure 4c). Liu et al. prepared highly sensitive NO_2_ sensors based on Pt-loaded In_2_O_3_ mesoporous nanofibers, which were synthesized by electrospinning, followed by a facile reduction method [75]. The In_2_O_3_ nanofibers functionalized with small Pt nanoparticles demonstrated superior NO_2_ sensing properties at room temperature, achieving a low LoD down to 10 ppb (R_g_/R_a_ = 2.8). In ambient conditions, the well-dispersed Pt nanoparticles within the composite fibers efficiently promoted oxygen molecular dissociation, generating abundant surface oxygen species. Upon NO_2_ exposure, these Pt nanoparticles actively accelerated the chemical interactions between NO_2_ molecules and adsorbed oxygen species. Through chemical sensitization effects, the optimized Pt distribution significantly lowered the activation energy required for NO_2_ adsorption. Additionally, NO_2_ molecules preferentially accumulated near Pt-In_2_O_3_ interfacial regions due to the reduced energy barrier for electron transfer from the conduction band of In_2_O_3_ to adsorbed NO_2_. However, the oxygen species induced by “spillover effect” were open acceptors for gases without selectivity. Furthermore, functionalization with noble metals could be coupled with morphostructural control to synergistically boost the performance of gas-sensing layers. Kim et al. synthesized both bi-modal pores and Pt catalyst-loaded thin-walled SnO_2_ nanofibers by Ostwald ripening-driven electrospinning combined with a sacrificial templating route using polystyrene (PS) colloid and bioinspired protein (Figure 4d) [76]. The Pt-decorated porous SnO_2_ nanofibers showed a high response of 192 to 5 ppm acetone, which was a 37-fold enhancement over that of pure SnO_2_. In addition, the Pt-PS-SnO_2_ sensors demonstrated negligible cross-response to interfering analytes, and a low detection limit of 10 ppb. The significant enhancements could be attributed to the synergistic effect between porous tubular structures and the superior sensitization effect of Pt/PtO_x_, as shown in Figure 4e. Choi et al. proposed microporous WO_3_ nanotubes functionalized with Pd nanoparticles for enhanced hydrogen sensing performance [77]. The microporous thin-walled nanotube structures were obtained by introducing colloidal polystyrene (PS) particles to a shell solution of W precursor and poly(vinylpyrrolidone). Pd nanoparticles, synthesized by bio-inspired protein cages, were uniformly dispersed within the shell solution and subsequently on the WO_3_ nanotubes. During the adsorption of H_2_S on the sensitive layer, Pd could dissociate H_2_ molecules into H atoms on the surface of Pd nanoparticles. The hydrogen atoms migrated across the WO_3_ surface through the spillover mechanism, where they interact with chemisorbed oxygen species (O^−^, O_2_^−^), thereby enhancing the receptor function of the sensitive layer. Pd-functionalized macroporous WO_3_ nanotubes exhibited a very high H_2_ response of 17.6 at 500 ppm and a low limit of detection (10 ppm) with a short response time of 25 s.

In addition to the catalytic effect of noble metals, the formation of Schottky junctions between noble metals and metal oxides would promote the sensing performance. Hung et al. utilized Au nanoparticles to dope SnO_2_/ZnO nanofibers to enhance the H_2_S sensing properties [78]. Due to the higher work function of Au (5.1 eV) than that of SnO_2_ (4.9 eV) and ZnO (4.45 eV), Schottky junctions were formed at the interface of Au and metal oxides. When exposed to H_2_S, the Schottky barrier height was reduced, leading to the resistance of Au-doped SnO_2_/ZnO nanofibers decreasing significantly. Hence, the Au-doped SnO_2_/ZnO sensor exhibited larger response toward H_2_S than undoped SnO_2_/ZnO (73.3 vs. 13.3 @1 ppm). To probe the selectivity mechanism of the noble metal modification on metal oxides, Liu et al. synthesized various noble metal nanoparticles modified ultrafine In_2_O_3_ nanofibers (Au-, Ag-, and Pt-In_2_O_3_) using a simple electrospinning method, followed by a controlled thermal treatment [79]. The Au-In_2_O_3_, Ag-In_2_O_3_, and Pt-In_2_O_3_ sensors exhibited improved selectivity toward H_2_S, HCHO, and acetone, respectively. Then, DFT calculations were implemented to reveal the adsorption energy and energy band structures of different noble metals doped In_2_O_3_. The most stable adsorption configurations occurred when H_2_S, HCHO, and acetone gas molecules adsorbed on the surface of Au-, Ag-, and Pt-In_2_O_3,_ respectively. Therefore, the specific adsorption energies between the noble metal modified metal oxides and target gases, along with the “spillover effect” led to excellent specific selectivity.

In addition to conventional single noble metal modification for metal oxides, bimetallic modification strategies leveraging synergistic effects have recently garnered increasing research attention [80]. Sui et al. prepared 1D In_2_O_3_ nanofibers functionalized by the well-shaped and monodispersed AuPt nanocatalysts (diameter: ~9 nm) for highly sensitive gas sensors (Figure 4g) [81]. The introduction of Au into Pt-based nanocatalysts enabled precise modulation of noble metal electronic structures while optimizing structural parameters, including Pt-Pt interatomic distances and heterometallic coordination numbers. The atomic engineering resulted in ligand effects that lower Pt binding energies in the Au-Pt heterostructure, triggering a downshift in the d-band center position. Consequently, the AuPt bimetallic system exhibited superior electrocatalytic activity compared to monometallic Au or Pt counterparts, leading to enhanced sensitivity toward ozone and acetone (Figure 4h). Similarly, Zhao et al. reported a highly sensitive and selective chemiresistive sensor for acetone detection in exhaled breath, leveraging electrospun ZnFe_2_O_4_ nanofibers functionalized with Au@Pt core–shell nanospheres [82]. The Au@Pt-ZnFe_2_O_4_ achieved excellent sensing performance, including a low LoD of 30 ppb at a low operation temperature of 188 °C, and superior performance compared to pristine ZnFe_2_O_4_ nanofibers (response: 3.32 vs. 1.84 toward 0.5 ppm acetone). Enhanced sensing performance resulted from the synergistic effects: (1) oxygen vacancies introduced via compositional engineering created sufficient active sites and charge carriers; (2) the Au@Pt bimetallic structure induced Schottky junctions and catalytic activity, accelerating gas–solid interactions and lowering activation energy.

### 4.2. Gas Sensor Based on MO-MO Heterojunctions

Constructing heterojunctions with other metal oxides is one facile strategy to improve the gas-sensing performance of pure metal oxide nanofibers [83]. According to the conductivity type of metal oxides, metal oxide heterojunctions can be classified into p-n heterojunctions, p-p heterojunctions, and n-n heterojunctions. Due to the difference in Fermi energy level of metal oxides, upon contact, electrons would transfer from the metal oxide with a high Fermi energy level to that with a low Fermi energy level until the equilibrium was established, with the formation of an electron depletion layer at their interface [83]. In addition, when the heterojunction is formed at the interface of two distinct metal oxide materials, the structural mismatch of their lattice constants and crystal structures generates interfacial stress. And the stress induced the dislodgement of oxygen atoms from the lattice framework, resulting in the formation of oxygen vacancies at the interface [84]. The oxygen vacancies would facilitate the adsorption of gas molecules on the sensitive layer. Moreover, abundant heterojunctions amplified the effect of the carrier-concentration change during the gas–solid interaction, eventually promoting the sensor response [63]. Therefore, metal oxide heterojunction nanofibers exhibited remarkable gas-sensing performance.

**Figure 5:**
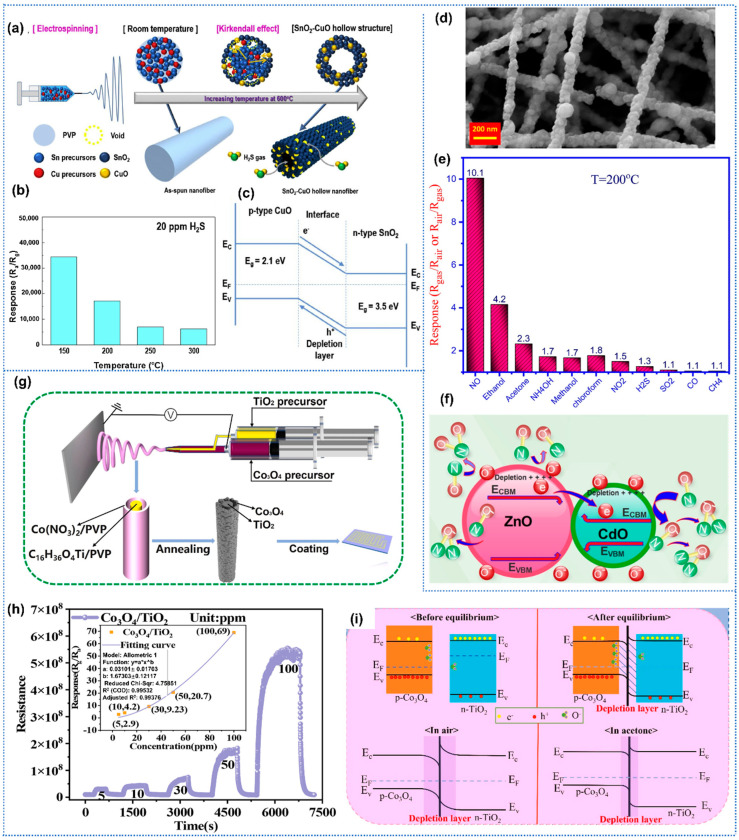
(**a**) Schematic process of SnO_2_-CuO hollow nanofibers by electrospinning; (**b**) Response of SnO_2_-CuO hollow nanofibers sensor toward H_2_S under different operation temperatures; (**c**) Schematic illustration of the p-n junction of SnO_2_-CuO, Reprinted with permission from Ref. [85] Copyright 2020, Elsevier. (**d**) SEM images of ZnO/CdO nanofibers; (**e**) Response of the ZnO/CdO sensor toward various gases; (**f**) schematic illustration of the mechanism of ZnO/CdO, Reprinted with permission from Ref. [87] Copyright 2019, Elsevier. (**g**) Schematic diagram of the synthesis procedure of Co_3_O_4_/TiO_2_ core–shell nanofibers using coaxial electrospinning; (**h**) Co_3_O_4_/TiO_2_ sensors to acetone (5–100 ppm) at 300 °C; (**i**) Gas-sensing mechanism of the Co_3_O_4_/TiO_2_, Reprinted with permission from Ref. [88] Copyright 2024, Elsevier.

Park et al. prepared porous CuO-SnO_2_ nanotubes by electrospinning for highly sensitive detection toward H_2_S [85]. The composite nanotubes with homogeneously distributed CuO nanoparticles within a tubular SnO_2_ structure were synthesized by electrospinning with a mixed solution of Sn and Cu precursors, followed by thermal treatment (Figure 5a). The CuO-SnO_2_ sensor exhibited similar sensing behavior with n-type SnO_2_ sensor toward H_2_S, indicating the host character of SnO_2_ in composites. The heterojunction sensors exhibited excellent H_2_S sensing performance, including a high response of 1395 with a short response time of 5.27 s to 5 ppm H_2_S at 200 °C, and remarkable selectivity (Figure 5b). The noticeably high sensitivity could be attributed to the formation of CuO-SnO_2_ p-n heterojunctions (Figure 5c). And the formation of CuS and then conversion to CuO on the surface of the CuO-SnO_2_ during the H_2_S adsorption and desorption process enhanced the selectivity of the sensing layer toward H_2_S. Similarly, Li et al. synthesized nanofibers of p-type NiO/n-type ZnO heterojunction for trimethylamine (TMA) detection by a facile electrospinning technique [86]. The effect of NiO content in composites on TMA sensing performance was investigated in detail. And the NiO/ZnO nanofibers with optimized component ratios exhibited significantly improved TMA sensing performance, including a high response of 892 to 100 ppm TMA, a low LoD down to 0.5 ppm, and short response/recovery times of 30/35 s. Naderi et al. reported a highly selective and sensitive electrospun ZnO-CdO nanofiber-based sensor for trace NO_2_ detection at an optimal temperature of 215 °C (Figure 5d) [87]. Based on the necklace-like ZnO-CdO n-n type heterojunction with a straddling bandgap, the sensor exhibited superior sensitivity over interfering gases (NO_2_, H_2_S, CH_4_) and volatile organic compounds (VOCs), achieving a remarkable response of 22.6 toward 33 ppm NO (Figure 5e). Moreover, the ZnO-CdO sensor presented rapid response/recovery times (47/1249 s to 3 ppm; 35/630 s to 33 ppm), long-term stability, and negligible humidity interference. The n-n heterojunction facilitated efficient charge transfer and surface reactions, while the ZnO-CdO interface regulated oxygen vacancy density and band bending, thus promoting gas adsorption dynamics (Figure 5f).

In addition to the above uniformly mixed metal oxides heterojunctions, the core–shell heterojunctions were also constructed to promote the gas-sensing performance of metal oxide nanofibers. Fu et al. reported an acetone sensor based on Co_3_O4-TiO_2_ core–shell nanofibers, which were fabricated easily by the coaxial electrospinning method (Figure 5g) [88]. In detail, they utilized a concentric spinneret to simultaneously process Co precursor Ti precursor, enabling the formation of core–shell nanostructured fibers with controlled interfacial architectures. The Co_3_O_4_-TiO_2_ sensors exhibited superior acetone sensing performance compared to pure TiO_2_ nanofibers and Co_3_O_4_ nanofibers sensors, including a high response of 20.7 toward 50 ppm acetone and a low detection limit of 0.5 ppm (Figure 5h). These enhancements could be attributed to the abundant adsorption and reaction sites provided by the core–shell nanostructures, the formation of p-n heterojunctions, and the good catalytic activity of Co_3_O_4_ (Figure 5i).

### 4.3. Gas Sensors Based on the Heterojunction of Metal Oxides-2D Materials

**Figure 6:**
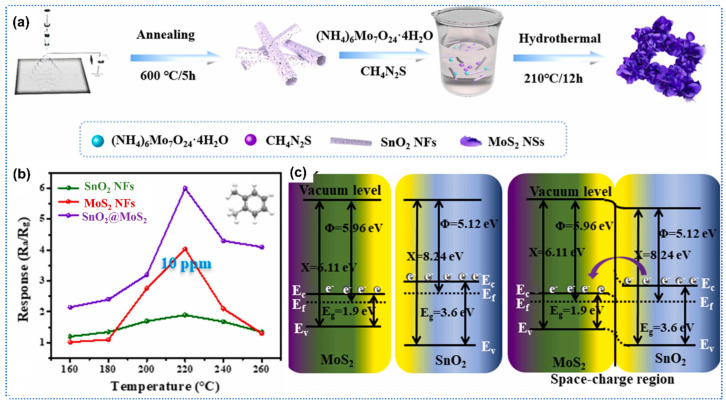
(**a**) Schematic diagram of MoS_2_-SnO_2_ synthesis process; (**b**) Response of SnO_2_, MoS_2_, and MoS_2_-SnO_2_ nanofibers toward xylene at different temperatures, (**c**) Schematic illustration of the sensing mechanism of the MoS_2_-SnO_2_ nanocomposites, Reprinted with permission from Ref. [92] Copyright 2023, Elsevier.

Recently, 2D materials have garnered considerable attention as potential candidates to functionalize metal oxides due to their large specific surface area, high carrier mobility, and strong gas adsorption ability [89]. Zhang et al. prepared conductive graphene oxide (GO)-WO_3_ composites nanofibers for acetone detection [90]. Thereinto, GO was added to the WCl_6_ precursor solution initially, then the GO-WCl_6_-PVP composites nanofibers were obtained by electrospinning, followed by calcination to synthesize GO-WO_3_ composites. Due to the modification of the GO, the GO-WO_3_ composites showed more porous structure compared to pure WO_3_, including higher specific surface area (17.01 vs. 11.40 m^2^/g), and larger pore sizes (29.36 vs. 7.95 nm). Porous structure would facilitate the adsorption and diffusion of gas molecules. Therefore, the GO-WO_3_ composites nanofibers showed a high response of 35.9 toward 100 ppm acetone at 375 °C, which was 4.3 times higher than that of pristine WO_3_ nanofibers. Meanwhile, the GO-WO_3_ composites exhibited short response/recovery times of 4/10 s toward acetone of 100 ppm. It is worth noting that with the increase in GO content in the composites, the response of GO-WO_3_ nanofibers would be decreased. This phenomenon resulted from the significant reduction in total sensor resistance due to the formation of conduction channel by interconnection of continuous GO layer, which led to the small magnitude of resistance change during the adsorption of gas molecules. Similarly, Wang et al. utilized the carbon materials including graphene, carbon nanotubes, and graphene oxide (GO) to sensitize SnO_2_ for enhanced formaldehyde sensing [91]. Due to the large specific surface area, rich functional groups, and the electric regulation effect of GO, GO-SnO_2_ composites nanofibers exhibited better sensing performance than pure SnO_2_, carbon nanotubes-SnO_2_, and graphene-SnO_2_ composites. The GO-SnO_2_ sensors showed a high response of 32 under exposure of 100 ppm HCHO at 120 °C, which was 4 times higher than that of pristine SnO_2_ nanofibers. In addition to graphene derivatives, transition metal chalcogenides (TMDs), such as MoS_2_ and WS_2_, as typical 2D materials, were widely used to functionalize metal oxides. Yang et al. developed hierarchical SnO_2_ nanofibers-MoS_2_ nanosheets core–shell nanocomposites for ultrasensitive xylene detection [92]. The synthesis process included two key steps: (1) electrospinning SnO_2_ nanofibers as the structural backbone, followed by (2) in situ hydrothermal growth of vertically aligned MoS_2_ nanosheets on the nanofiber surface to form heteroarchitected interfaces, as shown in Figure 6a. Benefiting from the high specific surface area of MoS_2_ nanosheets, the composites achieved a significantly enhanced specific surface area of 18.77 m^2^/g compared to 6.67 m^2^/g for pristine SnO_2_ nanofibers. The porous structure enabled the SnO_2_-MoS_2_ sensor to demonstrate remarkable performance at 220 °C, including a rapid response/recovery kinetics (21.5 s/60.4 s) and a high response of 2.35 to 100 ppm xylene, which was 4.7 times higher than that of pure SnO_2_ counterparts (Figure 6b). These enhancements originated from the synergistic effect of the following three aspects (Figure 6c): (1) abundant MoS_2_-SnO_2_ heterojunctions facilitating efficient charge transfer, (2) MoS_2_-induced oxygen vacancy defects providing more active adsorption sites, and (3) sufficient gas diffusion pathways through the hierarchical 3D nanostructure. Zhu et al. prepared hierarchical heterojunctions of WS_2_ nanosheets/ In_2_O_3_ nanofibers for an efficient detection of formaldehyde [93]. The In_2_O_3_ nanofibers were prepared by electrospinning, and then the WS_2_ nanosheets were grown on the In_2_O_3_ nanofibers by in situ hydrothermal reaction. The WS_2_-In_2_O_3_ composites possessed a high specific surface area of 84.9 m^2^/g, which significantly surpassed that of pure In_2_O_3_ (9.6 m^2^/g). Moreover, the WS_2_-In_2_O_3_ heterojunctions would enhance charge transfer and provide more active sites for gas adsorption. Therefore, the WS_2_-In_2_O_3_ composites demonstrated a high response of 12.6 to 100 ppm HCHO at 140 °C, which was two times higher than that of the pure In_2_O_3_ sensor.

### 4.4. Gas Sensors Based on Metal-Ion-Doped Metal Oxides

Metal ion doping serves as a dual-functional strategy to synergistically enhance surface lattice defects and oxygen vacancy concentrations, while doping sensitization introduces new donor/acceptor states through defect engineering, thereby elevating free carrier density and improving charge transport efficiency [94]. Electrospinning enables in situ metal ions doping within polymer-templated nanofibers, where spatial confinement effects and precursor solution viscosity regulate uniform metal distribution. This controllable doping approach enables precise stoichiometric engineering of metal-metal oxides, optimizing both gas adsorption sites and interfacial charge transfer kinetics.

**Figure 7:**
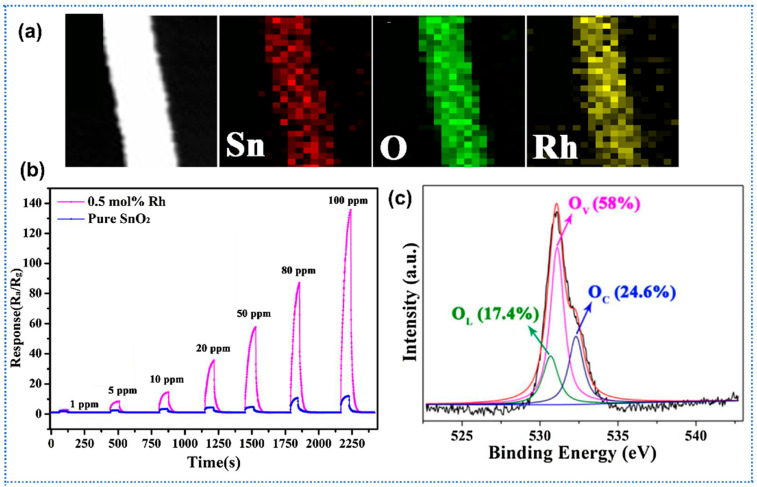
(**a**) EDS elemental mapping images of Rh-doped SnO_2_ nanofibers; (**b**) Response of sensors based on pure SnO_2_ and Rh-doped SnO_2_ nanofibers toward acetone of 1–100 ppm at 200 °C; (**c**) O 1s XPS of Rh-doped SnO_2_ nanofibers, Reprinted with permission from Ref. [98] Copyright 2018, Elsevier.

Wu et al. reported a systematic investigation of Cu-doped α-Fe_2_O_3_ semiconductor nanofibers synthesized via electrospinning for selective NO_2_ gas sensing at ppm levels [95]. By varying the Cu molar ratio in precursor solutions, the study revealed a unique phenomenon of p-to-n-type semiconducting transition in α-Fe_2_O_3_ nanofibers when Cu molar ratios exceeded 0.23, driven by the formation of CuFe_2_O_4_ spinel phases via cation substitution. The Cu-doped α-Fe_2_O_3_ fibers exhibited significantly shortened response time (up to 40% reduction at 33 ppm NO_2_) compared to undoped α-Fe_2_O_3_. Wang et al. prepared Ni-doped ZnO electrospun nanofibers for enhanced C_2_H_2_ gas sensing [96]. Through electrospinning followed by calcination, Ni doping (up to 5 at%) preserved the hexagonal wurtzite structure of ZnO nanofibers while introducing lattice distortions and intrinsic defects. Notably, 5 at% Ni-doped ZnO exhibited a sixfold increase in sensitivity (16.9 vs. 2.6 for undoped) toward 2000 ppm C_2_H_2_ at 250 °C, attributed to Ni^2+^-induced oxygen vacancy modulation and facilitated charge transfer. The hierarchical structure of nanofibers (diameters ~100 nm) displayed rapid response/recovery times (~5/10 s). Wu et al. utilized Al-doping to induce the formation of oxygen vacancy for promoting the gas-sensing performance of SnO_2_ nanofibers [97]. The hierarchical mesoporous Al-doped SnO_2_ nanofibers were prepared by a simple electrospinning method and annealing. Due to the substitution of Sn^4+^ by Al^3+^ in SnO_2_ lattice, large amount of oxygen vacancies generated with the doping of Al. The oxygen vacancy of SnO_2_ changed as the function of Al/(Al + Sn) ratio and the oxygen vacancy exhibited a maximum value with the ratio of 8%. Meanwhile, the sensor showed the maximum response of 7.82 toward formaldehyde (1 ppm) when the Al/(Al + Sn) ratio was 8%. Kou et al. reported a highly sensitive acetone sensor based on rhodium (Rh)-doped SnO_2_ electrospun nanofibers [98]. Utilizing electrospinning to fabricate uniform SnO_2_ nanofibers (~150 nm diameter) and subsequent calcination (Figure 7a), 0.5 mol% Rh doping SnO_2_ achieved a 9.6-fold enhancement in response (R_a_/R_g_ = 60.6) to 50 ppm acetone at 200 °C compared to undoped SnO_2_ (R_a_/R_g_ = 6.3) (Figure 7b). Rh doping induced lattice contraction, and thus reduced the crystallite size of SnO_2_ (from 9.85 nm to 6.56 nm). In addition, the Rh doping induced more oxygen vacancies (O_v_) than pure SnO_2_, which could supply more active sites for the gas reaction and adsorption, thereby enhancing the response (Figure 7c). Furthermore, the Rh-doped SnO_2_ sensor exhibited exceptional selectivity towards acetone over ethanol and other interfering gases, with short response/recovery times of 2/64 s.

Above reports exhibited the effective enhancement strategies for the metal oxide nanofibers, as shown in Table 1. However, current research primarily focused on enhancing the sensitivity of sensitive materials, and the proposed metal oxide functionalization method still suffered from high operation temperatures, which limits the further utilization of metal oxide nanocomposite fibers. In addition, the introduction of hydrophilic materials, such as CuO and NiO, would facilitate the adsorption of water molecules, thus reducing the response of metal oxide nanofibers [99].

**Table 1:** Gas-sensing performance based on metal oxide composites.

Material		Gas	S	OT	t_res_/_trec_	LOD	S_F_/S_N_	Ref.
Noble meta-doped	Pt-SnO_2_	acetone (2 ppm)	31.2 ^a^	150 °C	13/25 s	100 ppb	10	[74]
	Pd-WO_3_	H_2_ (500 ppm)	17.6 ^a^	450 °C	25 s/-	10 ppm	3.6	[77]
	Pt-PS-SnO_2_	Acetone (5 ppm)	192 ^a^	350 °C		10 ppb	37	[76]
	Au-ZnO/SnO_2_	H_2_S (1ppm)	73.3 ^a^	350 °C	36/786 s	0.1 ppm	7	[78]
	Au-In_2_O_3_	H_2_S (1 ppm)	13.6 ^a^	300 °C	35/108 s	50 ppb	3.5	[79]
	Ag-In_2_O_3_	HCHO (1 ppm)	12.6 ^a^	300 °C	19/24 s	8 ppb	4.1	[79]
	Pt-In_2_O_3_	acetone (1 ppm)	17.9 ^a^	300 °C	22/28 s	20 ppb	4.0	[79]
	Pt-In_2_O_3_	NO_2_ (1 ppm)	23.9 ^a^	RT	-/-	20 ppb	2	[75]
	AuPt-In_2_O_3_	acetone (50 ppm)	7.1 ^a^	240 °C	-/-	10 ppm	3.1	[81]
	Pd-Co_3_O_4_/ZnO	ethanol (200 ppm)	59 ^a^	240 °C	6/12 s	1 ppm	2	[100]
	Ag-SnO_2_	acetone (50 ppm)	40 ^a^	160 °C	6/10 s	5 ppm	-	[101]
	AuPt-ZnFe_2_O_4_	acetone (0.5 ppm)	3.32 ^a^	188 °C	33/28 s	30 ppb	1.8	[82]
MO-MO heterojunction	Co_3_O_4_-TiO_2_	acetone (50 ppm)	20.7 ^a^	300 °C	68/18 s	500 ppb	7	[88]
	NiO-ZnO	TMA (100 ppm)	892 ^a^	260 °C	18/20 s	0.5 ppm	9	[86]
	CuO-CdO	NO (33 ppm)	22.6 ^a^	215 °C	35/630 s	1.2 ppm	2.4	[87]
	SnO_2_-CuO	H_2_S (5 ppm)	1395 ^a^	150 °C	5.27 s/-	2 ppm	-	[85]
	ZnO-SnO_2_	NH_3_ (100 ppm)	60.41% ^b^	RT	70/23 s	10 ppm	-	[102]
	ZnO-In_2_O_3_	ethanol (100 ppm)	31.9 ^a^	225 °C	3/21 s	0.2 ppm	2	[103]
	ZnO-CuO	H_2_S (10 ppm)	4489.9 ^a^	150 °C	-/-	1 ppm	-	[104]
	NiO-SnO_2_	toluene (50 ppm)	11 ^a^	330 °C	11.2/4 s	50 ppm	10	[105]
	GO-SnO_2_	HCHO (100 ppm)	32 ^a^	120 °C	66/10 s	0.5 ppm	4	[91]
	MoS_2_-SnO_2_	xylene (100 ppm)	23.5 ^a^	220 °C	21.5/60.4 s	0.5 ppm	4.7	[92]
MO-2D material heterojunciton	GO-WO_3_	acetone (100 ppm)	35.9 ^a^	375	4/10 s	20 ppm	4.3	[90]
	WS_2_-In_2_O_3_	HCHO (100 ppm)	12.6 ^a^	140	30 s/43 s	1 ppm	2.0	[93]
	rGO-ZnO	H_2_ (0.1 ppm)	866 ^a^	400	210/234 s	0.1 ppm	-	[106]
	rGO-In_2_O_3_	NH_3_ (15 ppm)	23.37 ^a^	RT	17/241s	44 ppb	10	[107]
	rGO-ZnFe_2_O_4_	H_2_S (1 ppm)	147 ^a^	350	10 s/-	0.14 ppb	1.5	[108]
Ions-doping	Rh-SnO_2_	acetone (50 ppm)	60.6 ^a^	200	2/64 s	1 ppm	9.6	[98]
	Ni-ZnO	C_2_H_2_ (2000 ppm)	16.9 ^a^	250	5/10 s	100 ppm	6.5	[96]
	Cu-Fe_2_O_3_	NO_2_ (50 ppm)	2.0 ^a^	300	118/258 s	5 ppm	-	[95]
	Al-SnO_2_	H_2_ (100 ppm)	7.7 ^a^	340	3/2 s	10 ppm	2.5	[109]
	Al-SnO_2_	formaldehyde (1000 ppb)	7.82 ^a^	240	-/-	100 ppb	1.33	[97]
	Ni-ZnO	H_2_S (50 ppm)	474 ^a^	215	50/124 s	1 ppm	23.7	[110]
	Ce-ZnO	acetone (100 ppm)	20.3 ^a^	300	10/9 s	10 ppm	2	[111]

^a^ S = R_a_/R_g_ or R_g_/R_a_, ^b^ S = (R_a_ − R_g_)/R_a_ × 100% or (R_g_ − R_a_)/R_g_ × 100%.

## 5. Gas Sensors Based on Carbon Nanofibers (CNFs)

Carbon materials (carbon nanotubes, graphene, carbon nanofibers), serving as novel gas-sensitive materials, have earned significant attention in gas detection [112]. Thereinto, carbon nanofibers, as one weak p-type semiconductor, possess a high aspect ratio, sufficient adsorption sites, and high electrical conductivity [113]. Hence, carbon nanofibers were seen as the potential gas-sensitive materials to realize the high sensitivity at room temperature. Generally, carbon materials exhibited similar gas-sensing performance with p-type semiconductors, where their resistance decreased upon exposure to oxidizing gases and increased upon exposure to reducing gases. Compared to metal oxides electrospinning nanofibers, electrospun CNFs were less investigated due to their weak response, sluggish adsorption/desorption process, and poor selectivity, which frustrated their further application [114].

**Figure 8:**
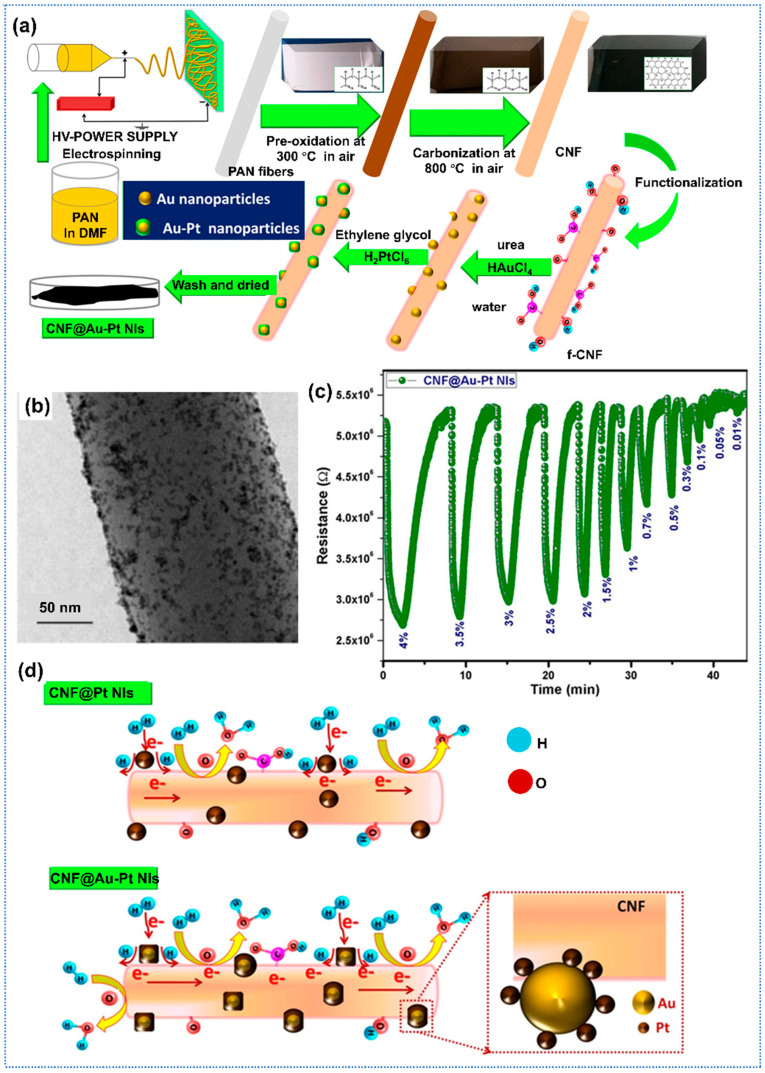
(**a**) Synthesis process of the Au-Pt doped carbon nanofibers; (**b**) SEM images of Au-Pt doped carbon nanofibers; (**c**) Dynamic response of Au-Pt doped carbon nanofibers toward H_2_; (**d**) Gas-sensing mechanism of noble metal doped carbon nanofibers to H_2_, Reprinted with permission from Ref. [115] Copyright 2020, The American Chemical Society.

Recent advancements in carbon-based gas sensors have demonstrated significant improvements in sensitivity and performance through structural and chemical modifications. Regarding noble metal modification, G. Nair et al. proposed bimetallic functionalized CNFs for the highly sensitive detection of H_2_ [115]. The composite material was synthesized via electrospinning followed by chemical reduction, resulting in uniformly distributed Au–Pt nanoislands (NIs) on acid-functionalized CNFs (Figure 8a). Figure 8b presented the TEM images of CNFs doped with Au-Pt nanoislands. The Au-Pt-CNFs exhibited superior hydrogen sensing performance, detecting trace-level H_2_ (0.01–4%) with a rapid response time (6.6 s) and recovery time (18 s), outperforming pure CNFs (Figure 8c). As for the enhanced gas-sensing mechanism, the adsorption kinetics studies aligned with the Langmuir model, demonstrating the dissociative H_2_ adsorption at Au-Pt active sites. In situ Raman spectroscopy revealed reduced structural disorder during H_2_ exposure, indicating the transfer of electrons from Pt to Au and CNFs, which enhanced the response of sensors (Figure 8d). In addition, the integration of Au mitigated irreversible Pt hydride formation, enhancing desorption and stability. Similarly, Nair et al. proposed one highly sensitive, flexible H_2_ gas sensor based on Ni/Pt-functionalized carbon fibers [116]. The Ni/Pt-CNFs sensors exhibited a superior response of 50% to H_2_ of 2%. Furthermore, the flexible sensor demonstrated a negligible decrease in response with mechanical stress under flat (21%) and bent (17%) states. Additionally, the baseline resistance of the sensor remains virtually unchanged after 100 successive bending cycles, due to the high aspect ratio of the carbon nanofiber networks, which allowed for a long bending path, indicating excellent mechanical robustness.

In addition to the noble metal modification, constructing heterojunctions with 2D materials, metal oxides, and other carbon materials was one facile strategy to improve the sensing performance of carbon nanofibers. Cha et al. utilized one 2D WS_2_ to functionalize CNFs for highly sensitive detection of NO_2_ at room temperature [117]. The WS_2_-CNFs were prepared via copolymer-electrospinning using two immiscible polymers, thereinto poly(styrene-acrylonitrile) (SAN) serving as sacrificial templates and poly(acrylonitrile) (PAN) serving as the carbon matrix, which enabled the creation of hierarchically porous CNFs with axial tubular channels, offering a high specific surface area (41.4 m^2^/g) and enhanced gas permeability. Moreover, monolayered WS_2_ nanoflakes (thickness ~0.3 nm) were uniformly embedded with edge-rich configurations on the CNF surface, leveraging their high d-orbital electron density to strengthen NO_2_ adsorption. The WS_2_-CNFs exhibited a notable response (15% at 1 ppm NO_2_) and excellent selectivity, attributed to synergistic effects between the WS_2_ edge sites and the porous conductive network. Wang et al. prepared an MoS_2_-carbon nanofibers network sensor by electrospinning and followed thermal treatment [118]. The MoS_2_-CNFs sensors exhibited a high response of 5.82% toward methane, which was 16 times higher than that of the pure CNFs. The introduction of n-type MoS_2_ nanoparticles not only increased the specific surface of the sensing layer and active sites, but also formed the heterojunction between carbon nanofibers, facilitating charge transfer between MoS_2_ and carbon nanofibers.

As for the modification of metal oxides, Lee et al. prepared carbon nanofibers decorated with WO_3_ using a single co-electrospinning process [119]. By leveraging phase-separated polymer solutions (PAN as the carbon matrix and PVP as the sacrificial template), the diameter of carbon nanofibers was controlled precisely over 40–130 nm, exhibiting a high specific surface area of 147–276 m^2^/g. The WO_3_ nanonodules (12–20 nm) were uniformly distributed on the CNF surface, forming p-n junctions which facilitated charge transfer upon NO_2_ adsorption, leading to a high response of 36% to 1 ppm of NO_2_. In addition, UV irradiation (75 mW cm^−2^) was employed to reduce recovery time from a few days to just 7 min. Similarly, Tang et al. prepared one flexible NH_3_ sensor based on ZnO nanoparticles decorated carbon nanofibers by electrospinning, peroxidation, oxidation, and carbonization [120]. The response of ZnO-CNFs toward NH_3_ of 50 ppm was 12.3%, which was ten times higher than that of pure CNFs. However, the NH_3_-sensing performance of ZnO-CNFs dropped significantly when the humidity exceeded 50%RH, indicating the poor humidity-resistant property.

Lee et al. report one synergistic strategy to improve the gas-sensing performance of carbon fibers in the aspect of pore structure, conductivity, and surface modification [55]. In this study, electrospun carbon nanofibers derived from polyacrylonitrile (PAN) were integrated with carbon black (CB) additives to enhance electrical conductivity, forming an internal conductive network critical for efficient electron transfer. Subsequent chemical activation using potassium hydroxide (KOH) generated a hierarchical porous structure, amplifying the specific surface area by approximately 100-fold (up to 2200 m^2^/g) and optimizing gas adsorption capacity. Surface fluorination further introduced electronegative functional groups (C-F bonds), which enhanced the selective attraction of polar gas molecules such as NO and CO. The synergistic effects of the modifications, including CB-enhanced conductivity, KOH-induced micro/mesopores, and fluorination-driven surface reactivity, synergistically improved sensor sensitivity by fivefold. Notably, the prepared sensors exhibited twice the sensitivity to NO compared to CO, attributed to its stronger electron localization and reactivity. This work underscored the potential of combining conductive additives, pore engineering, and surface functionalization to develop high-performance carbon-based sensors for environmental monitoring. To achieve a balance between high specific surface area and robust mechanical stability in carbon nanofibers, the mechanical stability can be effectively enhanced by optimizing stabilization and carbonization temperatures [121,122], as well as incorporating nanomaterials (such as carbon nanotubes and graphene nanosheets [123,124]) for precursor optimization.

The above modification strategy for the carbon nanofibers promoted the response significantly at room temperature, as shown in Table 2. It is worth noting that the carbon nanofiber-based sensors still suffered from the sluggish adsorption/desorption kinetics at room temperature, always taking considerable minutes to reach a steady state, which was significantly inferior to that of metal oxide gas sensors. Hence, developing more advanced research methodologies is critical to shorten response/recovery times of carbon nanofibers. Meanwhile, carbon nanofibers as hydrophilic materials, humidity would affect the gas-sensing performance of carbon nanofibers [125]. Shooshtari et al. reported the effect of humidity on the organic gases sensing performance of carbon nanotubes at room temperature [126]. The response to target gas decreased with the humidity increasing from 10% to 80%RH, indicating the unreliability of carbon nanofibers sensors under various humid environments.

**Table 2:** Gas-sensing performance of electrospinning carbon nanofibers gas sensors.

Material		Gas	S	OT	t_res_/_trec_	LOD	S_F_/S_N_	Ref.
Metal doped	Au/Pt-CNFs	H_2_ (4%)	47% ^b^	RT	6.6/18 s	100 ppm	-	[115]
	Fe-CNFs	H_2_ (500 ppm)	0.35% ^b^	RT	193/97 s	50 ppm	-	[127]
	Ni/Pt-CNFs	H_2_ (100 ppm)	13% ^b^	RT	32/72 s	100 ppm	-	[116]
Heterojunction	ZnO-CNFs	NH_3_ (50 ppm)	12.3% ^b^	RT	5/18 s	10 ppm	10	[120]
	WO_3_-CNFs	NO_2_ (20 ppm)	7.5% ^b^	RT	-/600 s	1 ppm	-	[119]
	WS_2_-CNFs	NO_2_ (1 ppm)	15% ^b^	RT	223.8 s/-	0.2 ppm	1.5	[117]
	MoS_2_-CNFs	CH_4_ (250 ppm)	5.82% ^b^	RT	-/-	10 ppm	16	[118]
	CB -CNFs	NO (50 ppm)	10% ^b^	RT	-/-	-	5	[55]
	fluorinated MWCNTs-CNFs	NO (50 ppm)	8% ^b^	RT	-/-	-	8	[128]

^b^ S = (R_a_ − R_g_)/R_a_ × 100% or (R_g_ − R_a_)/R_g_ × 100%.

## 6. Gas Sensors Based on Conductive Polymer (CP) Nanofibers

The tendency for agglomeration and non-uniform distribution during the synthesis of conductive polymers significantly compromises the accessibility of adsorption sites, thereby diminishing gas-sensing capabilities [129]. Moreover, their susceptibility to ambient degradation, unsatisfactory repeatability, and weak selectivity pose significant challenges for practical application [130]. To address limitations, advanced electrospinning engineering strategies have been pursued.

**Figure 9:**
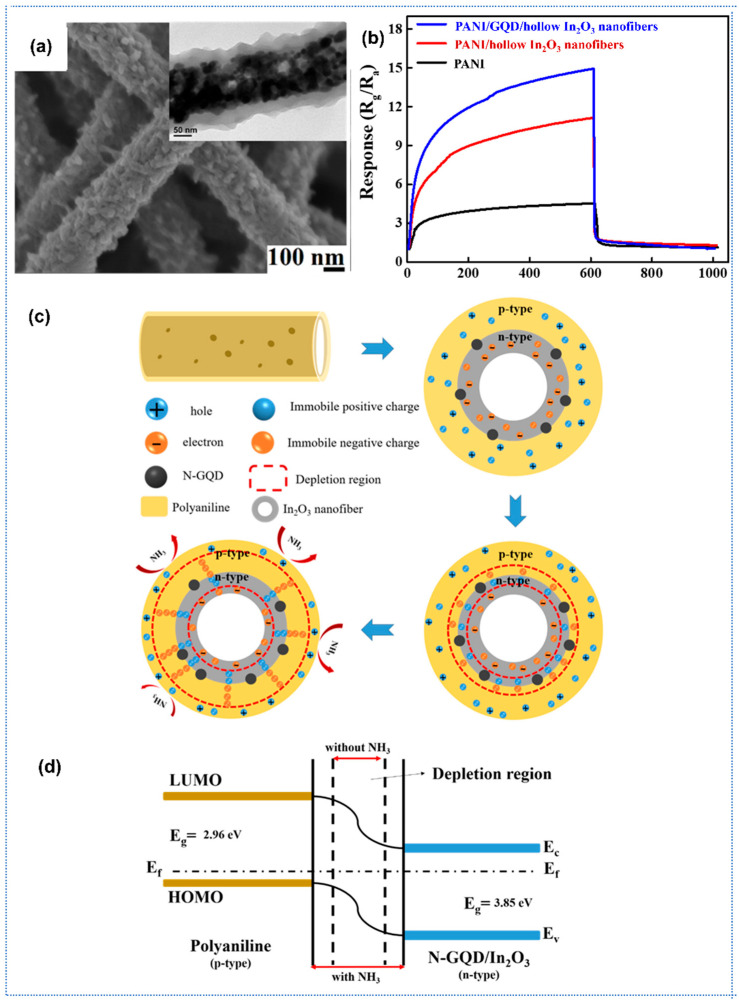
(**a**) SEM and TEM images of In_2_O_3_-PANI nanofibers, (**b**) response curves of PANI, In_2_O_3_-PANI, and N-GQD/In_2_O_3_-PANI composites, (**c**) schematic illustrations of the sensing mechanism and (**d**) energy band structure diagram of N-GQD/In_2_O_3_-PANI composites, Reprinted with permission from Ref. [133] Copyright 2021, MDPI.

Recently, composites of conducting polymers (CPs)-metal oxide for gas sensing have attracted considerable attention because the synergic effects of the CPs and metal oxides could significantly improve the gas-sensing performance compared with the pure CPs. Sharma et al. reported a highly sensitive H_2_ gas sensor based on SnO_2_-PANI composite nanofibers [131]. SnO_2_ nanofibers were initially synthesized by the electrospinning method as a framework. And then the SnO_2_-PANI nanocomposites were prepared by chemical oxidative polymerization of aniline on the substrate bearing SnO_2_ fibers. The SnO_2_-PANI sensor showed similar sensing behavior to pure PANI sensor, with a high response of 180 toward 5000 ppm H_2_ and a fast response time (<30 s) at room temperature. When the composites are exposed to H_2_, gas molecules would react with nitrogen atoms at imine sites of doped PANI, and these hydrogen molecules might form a bridge between nitrogen atoms of two polymer chains, which could enhance the conductivity of the sensing layer and thus improve the response. Beniwal et al. proposed SnO_2_-PPy nanocomposite for ultra-low ammonia concentration detection at room temperature [132]. Porous SnO_2_ nanofibers synthesized by electrospinning have a diameter in 70–150 nm, followed by vapor phase polymerization of pyrrole to develop SnO_2_-PPy composites The SnO_2_-PPy sensor exhibited a high response of 57% toward 100 ppb NH_3_ with fast response/recovery speed of 18/30 s at room temperature. Under ammonia exposure, the NH_3_ molecules would interact with the PPy and deprotonate the PPy sheath by decreasing the conducting charge carriers, leading to a decrease in conductance and thus an enhancement in response. Hong et al. reported a highly sensitive room-temperature ammonia (NH_3_) gas sensor based on a ternary composite of polyaniline (PANI), nitrogen-doped graphene quantum dots (N-GQDs), and hollow indium oxide (In_2_O_3_) nanofibers [133]. In their work, In_2_O_3_ nanofibers prepared by electrospinning and high-temperature calcination were coated with N-GQDs by hydrothermal reaction. Then, the N-GQD-coated hollow In_2_O_3_ nanofibers served as a core for the synthesis of polyaniline (PANI)/NGQD/ hollow In_2_O_3_ nanofiber ternary composites using in situ chemical oxidative polymerization (Figure 9a). The composite leverages the synergistic effects of high conductivity of PANI, catalytic activity of N-GQD, and the hollow nanostructure of In_2_O_3_ to achieve a remarkable response (R_g_/R_a_ = 15.2 @ 1 ppm NH_3_), and 4.4 times higher than that of pristine PANI (Figure 9b). The p-n heterojunction between PANI and N-GQD/In_2_O_3_ enhanced electron depletion layer dynamics (Figure 9c,d), while the hollow structure and N-doping increased specific surface area (102.1 m^2^/g) and active adsorption sites. Moreover, the composite sensor exhibited excellent selectivity to NH_3_ over interferents (methanol, ethanol), repeatability, fast response speed (<90 s), and a linear response across 0.6–2.0 ppm with a sensitivity of 23.95%/ppm (R^2^ = 0.994).

In addition to metal oxides, the introduction of noble metals, carbon-based materials, or ion doping is also an effective method to enhance the performance of polymer gas sensors. Han et al. proposed a methanol gas sensor based on the electrospun polymer/carbon nanotubes, which were prepared by mixing the poly (methyl methacrylate) and SWCNTs in solution with DMF and then using electrospinning technology [134]. The diameter of composite nanofibers ranged from 200 nm to 500 nm. The incorporation of carbon nanotubes improved the electrical percolation conductivity of polymers. When the polymer swelled due to methanol adsorption, the carbon nanotubes separated from each other and thus increased the resistance of the sensing layer, leading to an enhanced response. Huang et al. prepared magnesium-ion linked binder-less PEDOT:PSS nanofibers for highly sensitive detection toward organic gases dimethyl-formamide (DMF), dimethyl sulfoxide (DMSO), and propylene carbonate (PC) [135]. The binder-free PEDOT:PSS nanofibers were synthesized via electrospinning by introducing magnesium nitrate as a physical cross-linker, enabling controlled entanglement of rigid PEDOT:PSS chains without relying on traditional carrier polymers. The optimized Mg^2+^/PEDOT:PSS ratio (0.1) facilitated uniform nanofiber formation with diameters of 70–100 nm. XPS analysis demonstrated preferential migration of PEDOT to the fiber surface during electrospinning, creating a higher active surface area and enhanced porosity compared to bulk films. Impressively, the electrospun nanofibers exhibited superior sensing performance for DMF, DMSO, and propylene carbonate vapors at room temperature, achieving response magnitudes 2–3 orders of magnitude greater than pristine PEDOT:PSS films. The nice selectivity to DMF was attributed to its close solubility parameter match with PEDOT (23.54 vs. 24.7 MPa^1^/^2^), enabling efficient analyte-polymer interactions. The facile strategy for fabricating high-performance organic gas sensors through synergistic optimization of material morphology and interfacial chemistry offered promising avenues for non-invasive environmental monitoring and wearable biomedical applications. Saeb Mousavi et al. developed a flexible hydrogen sulfide (H_2_S) gas sensor based on electrospun polyaniline-polyethylene oxide (PANI-PEO) nanofibers doped with camphorsulfonic acid (HCSA), achieving room-temperature detection with high sensitivity, rapid response/recovery, and excellent stability [56]. The sensor, fabricated via a scalable electrospinning technique directly onto paper and polyimide substrates, exhibited a detection limit of 1 ppm H_2_S with response/recovery times of 120/250 s. The HCSA-doped PANI-PEO composite demonstrated superior selectivity toward H_2_S against interfering gases (NO_2_, acetone), while long-term stability tests revealed 4.7% sensitivity degradation over 45 days. In addition, mechanical bending tests confirmed the excellent robustness, with <1% performance loss after 1000 cycles. Pang et al. proposed an NH_3_ gas sensors based on Ag-PAN-PANI nanofibers [136]. The PAN nanofibers were prepared by electrospinning, serving as templates, and then Ag/APNI were synthesized by in situ polymerization method assisted with light irradiation and grew on the surface of PAN nanofibers. The Ag-PAN-PANI sensor showed a low limit of detection of 200 ppb NH_3_ at room temperature with high response, short response/recovery time, and excellent repeatability. It should be noted that lots of Ag nanoparticles in composites would form a separate current channel, and the charge transferred to Ag would not affect the resistance of PANI of sensors significantly, leading to a decrease in sensors. In addition, it should be noted that the response of Ag-PAN-PANI sensor toward NH_3_ decreased with the increase of humidity from 11 to 97%. Moreover, the Ag-PAN-PANI sensor exhibited significant response degradation of 36% in 15 days, indicating that the long-term stability should be enhanced.

It could be found that the above method could promote the sensing performance of polymers nanofibers (Table 3), including ultralow limit detection, enhanced response, and selectivity. However, the polymer nanofibers were vulnerable to ambient humidity due to the existence of the proton effect, swelling effects, and the competitive target gas and H_2_O molecules, leading to a reduction in response [137]. Moreover, the sensitivity shift in the sensor based on polymers during long-term applications should be considered. Regarding calibration of sensors, calibrated transfer strategy and classifier integration could offset drift efficiently [69]. In addition, accelerating life test, including increasing operation temperature and humidity, promoting the adsorption/desorption cycle frequency, would evaluate the sensor’s reliability [138]. Moreover, novel encapsulation strategy, such as utilizing polyvinylidene difluoride (PVDF) [139] or MOF-membrane as gas barrier layers [140], would improve the long-term stability of sensors.

**Table 3:** Gas-sensing performance of electrospinning polymer nanofibers gas sensors.

Material		Gas	S	OT	t_res_/_trec_	LOD	S_F_/S_N_	Ref.
Heterojunction	SnO_2_-PPy	NH_3_ (0.1 ppm)	57% ^b^	RT	18/30 s	0.1 ppm	-	[132]
	SnO_2_-PANI	H_2_ (5000 ppm)	1.35 ^a^	RT	30 s/-	1000 ppm	-	[131]
	TiO_2_-PANI	NH_3_ (0.2 ppb)	1.8% ^b^	RT	-/-	50 ppt	1000	[141]
	TiO_2_-PANI	NH_3_ (50 ppb)	0.52 ^b^	22 °C	-/-	25 ppb	-	[142]
	NiFe_2_O_4_-PANI	NH_3_ (100 ppm)	30.8 ^b^	RT	15/21 s	250 ppb	3	[143]
	N-GQD/In_2_O_3_-PANI	NH_3_ (1 ppm)	15.6% ^b^	RT	-/-	0.6 ppm	4.4	[133]
	MoS_2_/SnO_2_-PANI	NH_3_ (100 ppm)	10.9 ^a^	RT	21/130 s	200 ppb	3.1	[144]
	rGO/SnO_2_-PANI	H_2_S (0.1 ppm)	9.1 ^a^	RT	81/78 s	50 ppb	2	[145]
	Ag/SnO_2_-PANI	H_2_ (1000 ppm)	2.2 ^a^	40 °C	16/24 s	500 ppm		[146]
Ions-doping	Mg-PEDOT:PSS	DMF (200 ppm)	1.12 ^a^	RT	-/-	50 ppm	-	[135]
	HCSA-doped PANI-PEO	H_2_S (1 ppm)	5% ^b^	RT	-/-	1 ppm	-	[56]
Noble metal doped	Ag-PAN-PANI	NH_3_ (100 ppm)	15 ^a^	RT	-/-	0.4 ppm	4.8	[136]

^a^ S = R_a_/R_g_ or R_g_/R_a_, ^b^ S = (R_a_ − R_g_)/R_a_ × 100% or (R_g_ − R_a_)/R_g_ × 100%.

## 7. Summary and Future Perspectives

Electrospinning technology offers a transformative method in the synthesis of gas-sensitive materials. This review initially introduced the prepared method, gas-sensing mechanism of electrospinning materials, including metal oxides, carbon, and polymer nanofibers. This review subsequently addressed the inherent limitations of pure nanofiber-based gas sensors, and summarized recent advancements in enhancing sensing performance through strategic structural modifications of nanofibers, including noble metal functionalization, heterojunction construction, and controlled ion doping. The functional strategies exhibited significant enhancement effects on the gas-sensing performance of electrospinning nanofibers. Corresponding enhancement mechanisms of composite nanofibers were also discussed in this review. The electrospinning composite nanofibers exhibited considerable promise in the development of advanced gas sensors with highly sensitive, low-energy consumption, and rapid response. Furthermore, the metal oxides nanofibers presented high sensitivity and rapid response/recovery speed, and nice stability. However, gas–solid reactions demand substantial activation energy and mandate oxygen involvement. Carbon and conductive polymers nanofibers gas sensors could react with the target gas without the involvement of oxygen at room temperature, leading to the great potential of application. Whereas, the reaction kinetics were weaker than that of metal oxides, resulting in long response/recovery times. In addition, the hydrophilicity of carbon nanofibers and the proton effect and swelling effects of conductive polymers would compromise their gas-sensing performance. Following sections would discuss some critical challenges frustrated their further applications in detail:

(1) Precise synthesis and scale-up production of electrospinning nanofibers remain inevitable obstacles. On one hand, electrospinning is susceptible to voltage, temperature, and humidity fluctuations, resulting in excessively broad fiber diameter distributions that compromise gas diffusion path consistency. On the other hand, the functional additives strategy, particularly for noble metal, suffers from particle agglomeration within fibers, impairing the exposure of active sites. To mitigate batch-to-batch variability in electrospun nanofibers, characterized by fluctuations in fiber diameter, morphology, and porosity that critically compromise sensor consistency, a systematic approach integrating solution standardization, closed-loop process control, scalability management, and performance correlation is essential [147,148]. Solution properties must be rigorously standardized by controlling viscosity and conductivity via real-time monitoring, optimizing solvent evaporation through binary systems, and adjusting surface tension to ensure stable Taylor cone formation. In addition, closed-loop process control requires dynamic voltage compensation scaled to needle density in multi-needle systems, strict environmental stabilization to balance solvent retention and polymer integrity, and adaptive collector distance adjustments based on real-time fiber diameter feedback to maintain optimal jet stretching. Moreover, scalability challenges could be addressed by adopting needleless methods or asymmetric needle layouts to minimize field interference, alongside in-line quality control using laser diffractometry and machine learning for predictive adjustment [147].

(2) The operation temperature is still too high for the metal oxide-based nanofibers. Metal oxides were the most widely investigated electrospinning gas-sensitive materials. And the operation temperature of sensors was successfully reduced to a certain extent by noble metal doping, constructing heterojunctions with 2D materials or metal oxides, and element doping. However, the operation temperatures of metal oxide-based sensors typically exceed 200 °C, as shown in Table 1. Hence, it is necessary to further lower the operating temperatures of metal oxide nanofibers gas sensors for excellent stability and less power consumption. To address this challenge, in addition to the surface engineering on gas sensitive materials, introducing external light irradiation or plasmonic heating on materials are effective strategy [149,150]. Nikfarjam et al. utilized UV irradiation to promote the H_2_ sensing performance of TiO_2_ nanofibers [151]. Under UV irradiation, the response was enhanced 10 times, reducing the response and recovery times by a factor of 3–6. Furthermore, the operation temperature was reduced from 290 °C to 170 °C. The promotion of gas-sensing performance under UV could be attributed to the reduction in activation energy between the TiO_2_ surface and H_2_. Moreover, the synergistic effect of light irradiation and Au plasmonic nanostructure could promote the sensing performance of sensors. Gogurla et al. proposed Au-ZnO plasmonic nanostructure for highly sensitive detection toward NO at room temperature [152]. The Au-ZnO exhibited a localized surface plasma resonance (LSPR), leading to strong absorption, scattering, and local field enhancement. Hence, the sensitivity was significantly enhanced due to the LSPR effect of Au nanoparticles under visible light illumination at room temperature.

(3) An anti-humidity strategy should be considered in the electrospinning nanofibers preparation. For metal oxide nanofibers, the doping of Ni and Cu would facilitate the adsorption of H_2_O on the sensitive layer, and thus decrease the active sites for target gases with reduced sensitivity. Moreover, carbon nanofibers as hydrophilic materials and humidity would affect the gas-sensing performance of carbon nanofibers. Regarding polymer nanofibers, such as PANI and PPy, proton effect and swelling effects would affect the reaction between the sensitive layer and target gases, thus resulting in the degradation of sensitivity. Recent reports proposed effective anti-humidity methods for gas-sensitive materials, including surface engineering (noble metal doping [153], element doping [154], modification with hydrophobic materials [155]), physical isolation [156], working parameters modulation [157], humidity compensation [158]. Hence, introducing a novel anti-humidity strategy in electrospinning nanofibers is one bright way for further application.

(4) The selectivity of electrospinning nanofibers merits further intensive investigation. The sensitivity enhancement has been achieved by the introduction of additives and adjusting the architectures in the above reports. However, the discrimination capability for target gases continues to demand enhancement, particularly in multi-component systems where competitive adsorption effects significantly compromise detection specificity. And more selectivity enhancement mechanisms need to be proposed. Moreover, integrating a sensor array with artificial intelligence algorithms is a promising strategy for detecting the target gas in mixed gases [159]. Pattern recognition methods including principal component analysis (PCA) [160], linear discriminant analysis (LDA) [161], fast Fourier transform (FFT) [162], discrete wavelet transform (DWT) [160], and artificial neural networks (ANN) [163] have been employed to detect target gas in mixture gases. Hence, the integration of functionalization strategies for sensitive materials with pattern recognition algorithms represents a promising strategy to enhance the selectivity of electrospun fiber-based sensors.

(5) The wide application of gas sensors based on electrospun fibers still faced the challenge of cost, regulatory hurdles, and compatibility with IoT platforms. Continuous optimization of functionalization strategies for sensing materials can effectively reduce sensor costs. For instance, when using noble metal-modified nanofibers, the adoption of single-atom catalysts significantly reduces noble metal loading, thereby lowering material costs [164]. Additionally, prioritizing environmentally benign solvents and non-toxic reagents during material synthesis ensures compliance with health and environmental regulations. Furthermore, implementing self-calibration models in IoT deployments counteracts temperature drift during field operation.
